# Recent advances and future prospects of iron oxide nanoparticles in biomedicine and diagnostics

**DOI:** 10.1007/s13205-018-1286-z

**Published:** 2018-06-01

**Authors:** N. V. Srikanth Vallabani, Sanjay Singh

**Affiliations:** Division of Biological and Life Sciences, School of Arts and Sciences, Ahmedabad University Central Campus, Navrangpura, Ahmedabad, Gujarat 380009 India

**Keywords:** Magnetic resonance imaging, Feraheme, Theranostics, Computed tomography, Cell labeling, Nanozymes, Magnetic separation

## Abstract

Superparamagnetic iron oxide nanoparticles (SPIONs) are considered as chemically inert materials and, therefore, being extensively applied in the areas of imaging, targeting, drug delivery and biosensors. Their unique properties such as low toxicity, biocompatibility, potent magnetic and catalytic behavior and superior role in multifunctional modalities have epitomized them as an appropriate candidate for biomedical applications. Recent developments in the area of materials science have enabled the facile synthesis of Iron oxide nanoparticles (IONPs) offering easy tuning of surface properties and surface functionalization with desired biomolecules. Such developments have enabled IONPs to be easily accommodated in nanocomposite platform or devices. Additionally, the tag of biocompatible material has realized their potential in myriad applications of nanomedicines including imaging modalities, sensing, and therapeutics. Further, IONPs enzyme mimetic activity pronounced their role as nanozymes in detecting biomolecules like glucose, and cholesterol etc. Hence, based on their versatile applications in biomedicine, the present review article focusses on the current trends, developments and future prospects of IONPs in MRI, hyperthermia, photothermal therapy, biomolecules detection, chemotherapy, antimicrobial activity and also their role as the multifunctional agent in diagnosis and nanomedicines.

## Introduction

IONPs possess unique properties, which are used to display several applications in biomedicine such as diagnostics, imaging, hyperthermia, magnetic separation, cell proliferation, tissue repair and drug delivery. Although nanostructures of iron, cobalt, and nickel are known to exhibit superparamagnetic properties and high magnetic susceptibility, IONPs such as magnetite (Fe_3_O_4_), hematite (α-Fe_2_O_3_) and maghemite (γ-Fe_2_O_3_), are the most studied magnetic nanoparticle type. Owing to this property, IONPs display aggregation behavior under the magnetic field, which can be suspended again as a stable suspension after removal of the external magnetic field. In addition, the better colloidal stability, biocompatibility, and persistence magnetic properties of IONPs make them an excellent candidate for biomedical applications (Huang et al. 2009; Isa Karimzadeh 2017).

Fe_3_O_4_ NPs differ from other IONPs due to the presence of both Fe^2+^ and Fe^3+^ combinations, where divalent ions are organized at the octahedral sites and trivalent ions are split across the tetrahedral and octahedral sites. However, α-Fe_2_O_3_ contains Fe^3+^ ions distributed at their octahedral sites and in case of γ-Fe_2_O_3_ (termed oxidized magnetite), Fe^3+^ cations are distributed in octahedral and tetrahedral sites along with Fe^2+^ cation vacancies located at octahedral sites (Wu et al. 2015b). Due to their feasible polymorphism and their electron hopping nature, these IONPs were classified as potential candidates in both biological and technical applications. In recent years, to enhance the use of IONPs in advanced applications/technologies NPs are altered by the creation of active layers supported by polymers, inorganic metal/metal oxides or bioactive molecules (Gupta and Gupta 2005). The surface engineering of IONPs can be achieved by several methods through layering a coating material over the iron oxide core, to form core–shell structure or the NPs are dispersed in a matrix to form the beads (Gupta et al. 2007). In addition, a Janus structure can be formed with one-half of IONPs and the rest with functional material, further, IONPs are embedded between two functional materials to form a shell–core–shell structure (Wu et al. 2015b).

Several methods like thermal decomposition, co-precipitation, sol–gel, microemulsion, hydro-thermal, sono-chemical, microwave, electrochemical and biosynthesis were evolved to synthesize IONPs (Huber 2005; Wu et al. 2015b). However, the relation between size, shape, and magnetism in IONPs plays a crucial role in exhibiting their properties. For instance, Fe_2_O_3_ and Fe_3_O_4_ NPs display different ferrimagnetism at room temperature. Further, IONPs tend to lose their dispersity after long-term due to aggregation of particles and their magnetism gets diminished due to oxidation in air. Thus different approaches are implemented to stabilize the NPs in an inert atmosphere and also to make them water-soluble at physiological pH, for applications in nanomedicine (Wu et al. 2015b).

For biomedical applications and in vivo studies, low toxicity, biocompatibility, biodegradability, long retention time, and magnetism to localize the IONPs at the target play a crucial role (Wu et al. 2015b). In diagnosis IONPs, acts as a probe in magnetic resonance imaging (MRI), Positron emission tomography (PET), near-infrared fluorescence (NIRF) imaging (Ju et al. 2017; Xie et al. 2010) and in biosensors for detection of biomolecules like glucose, proteins, urea, and uric acid (Chen et al. 2012; Wu et al. 2015a; Yu et al. 2009). Comparatively, IONPs gained attention in therapeutic nanomedicine ranging from cancer treatment to antimicrobial activity (Nehra et al. 2018; Patra et al. 2017). In theranostics, IONPs are applied as nano-carriers, for enhancing the drug activity in combination therapy (IONPs and chemotherapeutic drugs) or as hyperthermia agents (Ren et al. 2012). Therefore, in the present review article, we focused on the IONPs applications in the domains of diagnosis and therapeutics.

## IONPs as imaging probe

### Role of iron oxide nanoparticles as contrast agent in Magnetic Resonance Imaging

**Fig. 1:**
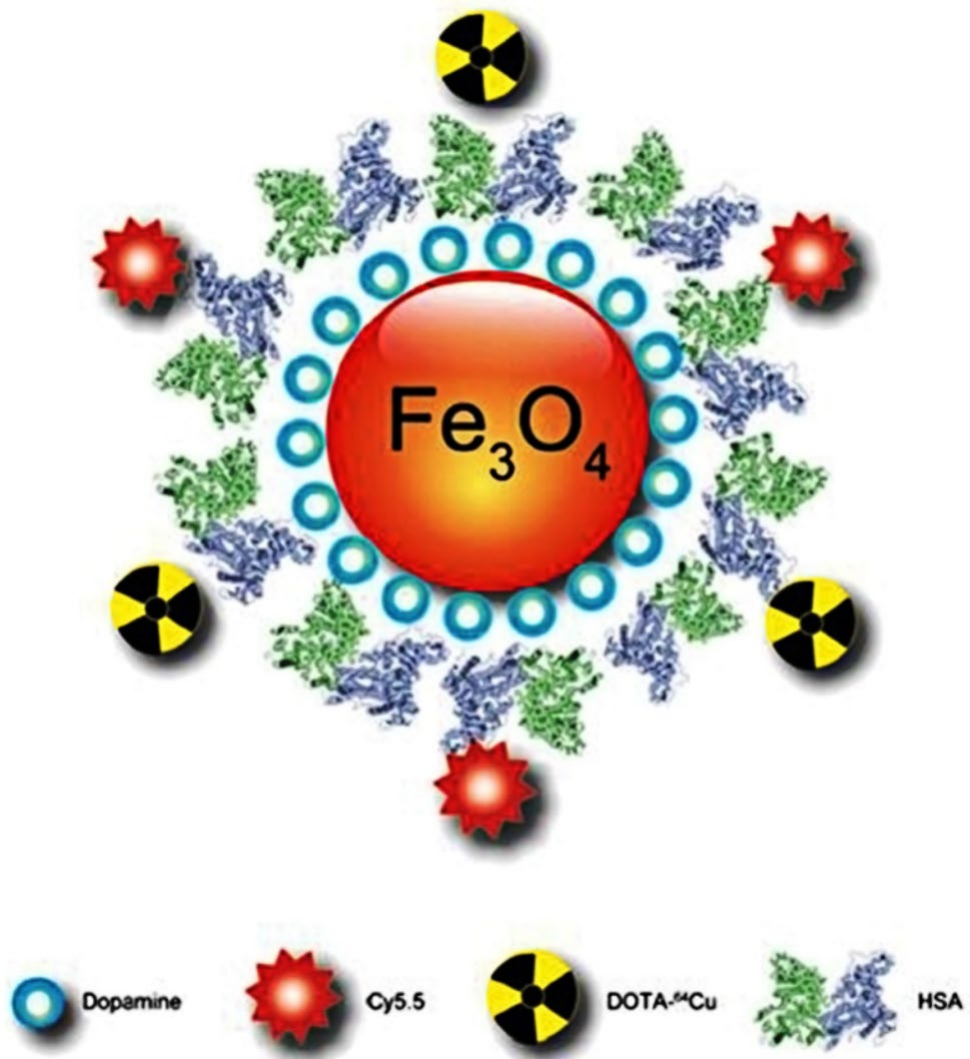
Schematic illustration of the multifunctional HSA-IONPs for triple active MRI/PET/NIRF imaging. The pyrolysis-derived IONPs were incubated with dopamine, after which the particles became moderately hydrophilic and could be doped into HSA matrices in a way similar to drug loading. Reprinted with permission from ref (Xie et al. 2010) Copyright (2010) Elsevier

MRI utilizes gradients of magnetic fields, radio waves and electric fields to elucidate the detailed internal structures of the body. MRI has a wide range of applications in detecting diseases or disorders of brain, heart, liver, blood vessels, and other vital organs. Recent research advancements have produced several excellent magnetic contrast agents such as gadolinium, superparamagnetic iron oxides, ultra-small (5–10 nm) superparamagnetic iron oxides, gadolinium doped carbon nanotubes, quantum dots embedded paramagnetic micelles and soft nanoparticles such as liposomes, perfluorocarbon emulsions, etc. (Emily and Waters 2008; Liu et al. 2017). Further, IONPs are prepared in combination with other NPs, proteins or dyes to achieve multiple applications in a single stage, for instance, MRI and immuno-histochemical staining of cancer cells was executed using ferrimagnetic H-ferritin (M-HFn) nanoparticles, dual MRI and CT imaging of tumor was simultaneously fulfilled by assembling gold (Au) nanocages‚ Fe_3_O_4_ NPs and triple functional iron oxide nanoparticles (Fig. 1) with Cy5.5 dye and ^64^Cu-DOTA chelate were developed for MRI, positron emission tomography (PET) and near-infrared fluorescence (NIRF) imaging (Cai et al. 2015; Wang et al. 2016; Xie et al. 2010).

Ferumoxytol (Feraheme) is one of the types of magnetic IONPs approved by the US Food and Drug Administration (FDA) for the treatment of patients suffering from iron deficiency with chronic kidney disease (CKD). It consists of a non-stoichiometric magnetite nanoparticle capped by poly glucose sorbitol carboxymethyl ether. Recently many imaging and therapeutic applications have been performed using ferumoxytol and the results suggest that it can be used in MR imaging with IONP cell labels for in vivo tracking of stem cells and also can be applied for non-invasive monitoring of stem cell therapies in pre-clinical and clinical setting (Castaneda et al. 2011). Moreover, studies delineated ferumoxytol-enhanced MRI was more sensitive for detection of early necrosis in tumor cells compared to other contrast agents (Aghighi et al. 2015). Reports also revealed that ferumoxytol gained interest to be used in renal failure patients as an alternative to gadolinium-based contrast agents for vascular MRI (Hope et al. 2015). Furthermore, as the feraheme is taken up by macrophages in liver, lymph nodes and spleen they can be explored for imaging macrophages, tumors, vascular lesions and other organs (Bashir et al. 2015; Vasanawala et al. 2016).

IONPs are a versatile class of material, which can be tuned to exhibit multifunctional applications. In one such attempt, Xie et al. (Xie et al. 2010) have developed dopamine modified IONPs, which was then encapsulated into human serum albumin (HSA) matrices. It was also shown that the HSA coated IONPs can also be labeled with two different dyes, ^64^Cu-DOTA and Cy5.5, to impart multiplexed imaging capability and tested them in a subcutaneous U87MG xenograft mouse model. Results revealed that a tri-modality imaging (including MRI, PET, and NIRF) was very much possible under ex vivo and in vivo experimental condition. HSA coating manifested longer blood circulation time, high extravasation and accumulation in targeted tissues and low uptake in macrophages in the nearby area of tumor.

**Fig. 2:**
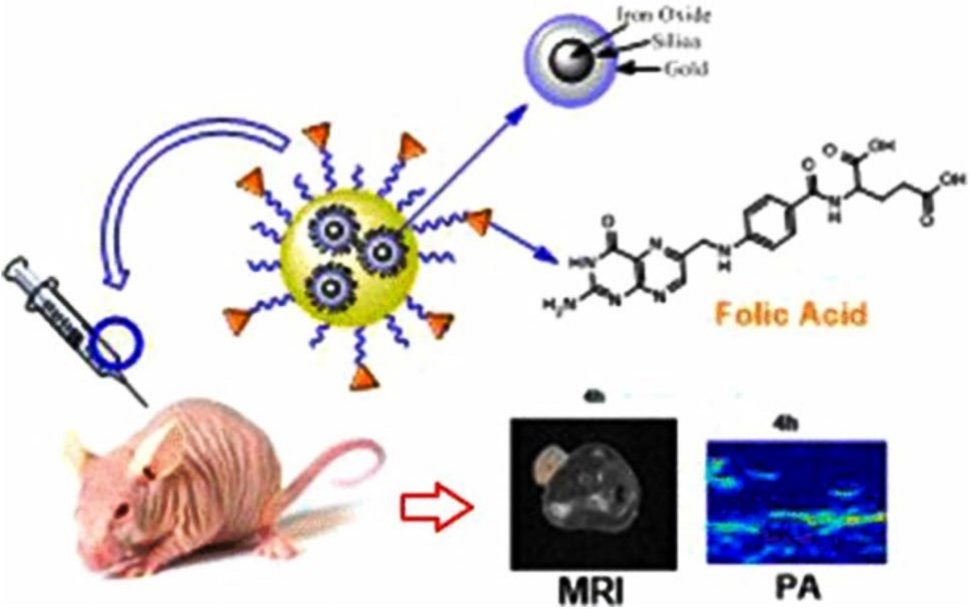
Fe_3_O_4_ NPs coated with inner silica and outer gold layers have been entrapped in polymeric micelles, decorated with folic acid moieties, and tested in vivo for photoacoustic and magnetic resonance imaging detection of ovarian cancer. Reprinted with permission from ref (Monaco et al. 2017) Copyright (2017) American Chemical Society

In the current context, image-guided photothermal therapy (PTT) is being looked as a promising alternative therapeutic modality to the most of the conventional methods. PTT is also expected to have potential to offer a better precision therapy alternative. Owing to the recent developments in material science, it is possible to synthesize the materials of our interest with variable size, shape, and composition. However, producing multicomponent materials with desired dimensions and stability still remains a challenge. Considering this, Ju et al. have developed a monodispersed composite material (Au–Fe_2_C Janus NPs) and used them as multifunctional cancer theranostics. These 12 nm particles exhibited a broad absorbance pattern under near infrared region, which lead to the generation of significant photothermal effect when irradiated with 808 nm laser light. This nanocomposite offered excellent optical and magnetic properties, which was found to be a promising method for triple-modal MRI/multispectral photoacoustic tomography (MSOT)/CT imaging both in in vitro and in vivo experimental models. Authors also modified this magnetic nanocomposite with HER2 affibody, which showed higher accumulation and deep tissue penetration in tumors than unconjugated particles (Ju et al. 2017). In another study by Monaco et al. developed a multi-layered nano-system composed of Fe_3_O_4_ NPs, coated with inner silica and outer Au layers. These NPs were entrapped into polymeric micelles and surface was conjugated with folic acid to offer them water solubility and target recognition. This novel nano-system was shown applications in targeting and magnetic resonance–photoacoustic based dual imaging modality. The significance of the complex system lies in having optical properties of gold NPs and a high dielectric constant of silica NPs ensuring strong light absorption capability (Fig. 2). Hence, they were utilized as a bimodal MRI-photoacoustic imaging (PAI) agent for imaging and detection of ovarian cancer (Monaco et al. 2017).

Malignant cells are characterized to have a significantly high rate of metabolism and glucose uptake. Utilizing this mechanism, cancer tissues are visualized under PET, involving high uptake of a radiolabelled glucose analog, [18F]-2-fluoro-2-deoxy-d-glucose (Lindholm et al. 1993). It is reported that the glucose transporter (Glut) proteins are found in the plasma membrane of mammalian cells, which facilitates the transport of glucose in the cytoplasm. Although there are several Glut proteins, however, Glut-1 has been shown to be involved in the high transportation of glucose in cancer cells (Singh 2017). Therefore, Glut proteins are considered as one of the suitable markers to selectively identify the cancer cells/tissues. The conjugation of Glut antibody with IONPs was shown to diagnose hemangioma (a condition in which noncancerous growths of blood vessels occur) through their MRI contrast imaging modality. The study was focussed on the differentiation of infantile hemangioma from vascular malformation, as Glut-1 is only expressed in cells of infant hemangioma (Sohn et al. 2015).

In general, IONPs acts as a probe for negative contrast (T_2_ contrast agents) due to their superparamagnetic behaviour and large magnetic moment. However, a typical dark signal produced in the T_2_ image can cause difficulty in distinguishing the areas of interest with calcium deposits, bleeding and glioma imaging. To overcome the limitation, Juan et al. synthesized citrate coated IONPs (C-ESION) with excellent positive (T_1_ imaging) and negative contrast through modulating the composition and coating thickness. In addition, authors demonstrated that the coating chemistry on NPs surface can change their relaxometric properties, which can be manipulated to generate particles with different contrasts. Results explained C-ESION120 displayed T_1_ weighed contrast and C-ESION140 showed T_2_ contrast signal characteristics. Hence, the modified coating enabled the maghemite NPs for T_1_ high-resolution MR angiography and also provided standard T_2_ contrast to utilizing them for a full range of applications in MRI (Pellico et al. 2017). Further, Ning et al. synthesized Gd-doped and PEG-coated IONPs (PEG-GdIO) having T_1_–T_2_ bimodal contrast ability and demonstrated the simultaneous T_1_–T_2_ contrast imaging in mice bearing glioma. Results revealed that after 1 h of NPs injection T_1_ and T_2_ weighted images showed brighter and darker contrast compared to pre-injection MR images. Moreover, the contrast enhancement was analyzed by contrast to noise ratio and significant improvement in signals was found for both T_1_ and T_2_ contrast images. Hence, it was concluded that PEG-GdIO NPs can be used as dual contrast agent for brain glioma detection (Xiao et al. 2014). Cha et al. modified Fe_3_O_4_ NPs (Fe_3_O_4_@GCP—Fe_3_O_4_ surface modified with glutathione, cyclodextrin and polymer) through polymer coating composed of β-cyclodextrin core and poly [2-(dimethylamino) ethyl methacrylate] arms and in association with reduced glutathione (GSH) as a model drug. The designed platform was expected for simultaneous diagnosis and treatment purposes. Authors suggested Fe_3_O_4_ NPs coated with these star polymers exhibited more GSH association compared to linear polymers and showed better stability in serum solutions. Further Fe_3_O_4_@GCP rendered low cytotoxicity and possessed enhanced T_1_ MRI characteristics. Results showed significant bright enhancement observed for T_1_ weighted images of liver visualization suggesting that the modified NPs can be used for diagnosis and treatment of chronic liver diseases (Cha et al. 2017).

### Use of iron oxide nanoparticles as contrast agent in computed tomography

CT is an X-ray based whole body imaging technique that combines series of computer processed X-ray images to construct the cross-sectional images of specific areas. CT is used to diagnose diseases or internal injuries in blood vessels, bones, soft tissues and other parts of the body. Clinically approved CT contrast agents for intravenous injection are iodinated small molecules or barium suspensions. Due to hypersensitive to iodinated contrast and renal impaired patients there is a need for better contrast agents. So, several nanoparticles including metallic, polymeric, liposomes, lipoproteins, micelles, and emulsions have been reported to yield better results as contrast agents for CT imaging (Cormode et al. 2014; Thomas et al. 2013) Carril et al. worked on 6 nm gold-coated Iron oxide glyco NPs and showed they can be effectively used for multimodal imaging in CT, MRI, and ultrasound (US) as contrast agents. Results explained that the increased gold coating on NPs surface was able to enhance the CT contrast through X-ray attenuation. Moreover, NPs with reduced size, sugar coating and negative surface charge allowed for long circulation time in blood and proved to be biocompatible (Mónica Carril 2014). Additionally, Naha et al. reported the synthesis of a composite consisting of bismuth–iron oxide nanoparticles (BION) with dextran coating. Data revealed that no cytotoxicity was observed in HepG2 (human liver cancer cell line) and BJ5ta (human fibroblast cell line) after 24 h incubation with NPs. In vivo CT imaging with optimized NPs concentration showed the contrast in heart and blood vessels indicating prolonged circulation half-life. Further, some contrast was observed in liver and spleen suggesting that the non-specific nanoparticle accumulation in these organs. It was concluded that NPs are biocompatible, biodegradable and possess strong X-ray attenuation characteristics, which can be used as a strong contrast agent for dual CT and MRI imaging (Naha et al. 2014). In another attempt, Perlman et al. indicated that IONPs can be used in ultrasonic computed tomography (UCT) for breast imaging. The imaging showed an improvement in contrast to noise ratio and can also serve as a pre-screening platform for disease diagnosis. Moreover, it was suggested that these NPs can be applied for multimodal MRI-ultrasound imaging purposes (Perlman and Azhari 2017). Reguera et al. designed a novel gold–iron oxide-based Janus magnetic-plasmonic NPs as contrast agents for imaging under CT, MRI, PAI, TEM, surface enhanced Raman spectroscopy (SERS) and optical microscopy. These complementary techniques allow obtaining maximum information and can serve as a multipurpose biomedical platform (Reguera et al. 2017).

### Iron oxide nanoparticles in positron emission tomographic imaging

PET is a nuclear imaging method that provides whole-body imaging and evaluates tissue and organ functions thus  enables quantification and localization of activity. However, it cannot reveal anatomical or morphological imaging. So to achieve advancements in imaging, CT, MRI or US can be combined to form a hybrid system such as PET/CT or PET/MRI for better resolution and anatomy of cells and tissues (Evertsson et al. 2017). de Rosales et al. reported the synthesis of a novel NPs system through bifunctional chelator dithiocarbamate–bisphosphonate conjugation to ^64^Cu and dextran coated IONPs for PET and MR imaging. Further, the labeling of clinically available IONPs (Endorem/Feridex) with ^64^Cu-based bifunctional chelator was performed and their dual-modality imaging was demonstrated in vivo in lymph nodes (Torres Martin de Rosales et al. 2011). Nahrendorf et al. conjugated a PET tracer ^64^Cu to dextran coated magneto fluorescent NPs to yield a tri-modality reporter (^64^Cu-TNP) for PET, MRI and fluorescence imaging. The capability of multimodal NPs was applied to detect macrophages in atherosclerotic plaques. Authors hypothesized that the in vivo PET signal correlates well with the inflammatory plaques observed by MRI, fluorescence imaging and flow cytometry (Nahrendorf et al. 2008). Xiaoqiang et al. developed a multifunctional nanocarrier functionalized with tumor targeting ligand, DOX-

conjugated and ^64^Cu labeled IONPs. The carrier provides targeted anticancer drug delivery and PET/MRI-based dual imaging modality of tumors expressing integrin α_v_β_3_. In vitro studies explained cRGD ligand (cyclic arginine-glycine-aspartic peptides) conjugated NPs exhibited more cellular uptake and tumor accumulation compared to free NPs shown by quantitative PET imaging and biodistribution analysis (Yang et al. 2011). A similar study was performed by Lee et al. to develop a bifunctional probe for PET and MR imaging. Polyaspartic acid coated IONPs were synthesized and conjugated with a cRGD ligand for integrin targeting and labeled with ^64^CuDOTA for PET analysis (Lee et al. 2008). Additionally, Chakravarty et al. synthesized ^69^Ge-labeled metal oxides by mixing IONPs with ^69^Ge ions. The simple method has an advantage in forming intrinsic radiolabelled NPs without any use of chelators. In addition, these NPs can be used for simultaneous PET and MRI imaging (Chakravarty et al. 2014) (Table 1).

**Table 1:** Role of iron oxide nanoparticles in various imaging modalities

Nanoparticle/material	Size (nm)	Applications/results	References
Ferumoxytol (Feraheme)	17–30	Treatment for anemia in renal failure patients Used in MR imaging for Stem cell tracking and macrophages. Showed high T_1_ signal near tumor necrosis regions (can be used for early necrosis detection). Can be effectively used for MR angiography in renal failure patients compared to gadolinium-based contrast agents	Aghighi et al. (2015), Bashir et al. (2015), Castaneda et al. (2011), Hope et al. (2015) and Vasanawala et al. (2016)
IONPs coated with HSA, Dopamine and labeled with ^64^Cu-DOTA and Cy5.5	15	Used for in vivo tri-modality imaging where MRI used for the study of particle distribution pattern. Under PET imaging showed better signal to noise ratio. NIRF used for both in vivo and ex vivo fluorescence-based imaging	Xie et al. (2010)
Fluorescent MNPs	10–40	Used for cell imaging (biological imaging)	García et al. (2018)
JNPs (Au-Fe_2_C Janus nanoparticles)	12	Applied for triple-modal imaging (in vivo and in vitro) Including MRI, CT, and PAI. Results showed that targeting of NPs was achieved by affibody conjugation (Au-Fe2C-Z_HER2:342_)	Ju et al. (2017)
Lipophilic Core − Shell Fe_3_O_4_@SiO_2_@Au (Fe_3_O_4_ coated with inner silica and Au outer layer)	157–222	Results showed targeting of cancer cells through folate receptors. Dual imaging capability for detection of ovarian cancer using MRI, and PAI	Monaco et al. (2017)
GLUT1-Fe_3_O_4_ NPs (Glucose transporter antibody conjugated Fe_3_O_4_ NPs)	10	Differentiation of infantile hemangioma from vascular hemangioma through MRI imaging was realized	Sohn et al. (2015)
C-ESION (Citrate coated IONPs)	3.5–4.5	For both T_1_ and T_2_ contrast imaging, where C-ESION120 used for T_1_-weighted angiography and C-ESION140 for T_2_-weighted MRI imaging	Pellico et al. (2017)
PEG-GdIO (PEGylated Gd-doped iron oxide NPs)	4.29–4.74	Showed simultaneous T_1_–T_2_ dual-modal MRI imaging and efficient diagnosis of brain gliomas	Xiao et al. (2014)
Fe_3_O_4_@GCP	10–22	Suitable for T_1_ MRI contrast where T_1_ weighted images of mice liver were capture with a signal intensity of ~ 1.2 times more compared to control	Cha et al. (2017)
Ultra-small IONPs	3–4	For T_1_ contrast imaging using MRI	Bao et al. (2018)
Fe_3_O_4_@Au@Glc/CO_2_H NPs (Gold-Coated Iron Oxide Glyco-nanoparticles)	6.1	Can be used as multimodal contrast agent for CT, T_2_ weighted MRI and US imaging	Mónica Carril (2014)
BION (Dextran coated bismuth–iron oxide nanohybrid)	5–15	Biocompatible and biodegradable NPs used for CT and T_2_ weighted MRI imaging	Naha et al. (2014)
Fe_3_O_4_	10	Used for ultrasonic breast imaging, also be applicable for multimodal MRI-ultrasound imaging	Perlman and Azhari (2017)
Gold-iron oxide NPs	20–50	Showed potential for multi-purpose imaging such as contrast agent for CT, T_2_ weighted MRI imaging and PAI	Reguera et al. (2017)
^64^Cu-IONPs (^64^Cu and dextran coated IONPs)	5	Showed better contrast for in vivo PET-MR imaging	Torres Martin de Rosales et al. (2011)
^64^Cu-TNP (IONPs as trireporter NPs)	20	Used for tri-modality NPs system (PET, MRI and fluorescence imaging) for direct detection of macrophages in inflammatory atherosclerosis	Nahrendorf et al. (2008)
cRGD-conjugated SPIO nanocarriers (cRGD-functionalized, DOX-conjugated, and ^64^Cu-labeled superparamagnetic iron oxide nanoparticles)	10	Multifunctional NPs for tumor targeting through conjugated cRGD (tumor targeting ligand) and quantitative PET-MRI imaging	Yang et al. (2011) and Lee et al. (2008)
Germanium-69-Labeled IONPs	10	Used for in vivo dual modality PET and MRI imaging	Chakravarty et al. (2014)

## Bio-sensing applications of iron oxide nanoparticles

### Role of Iron oxide nanoparticles as nanozymes

**Fig. 3:**
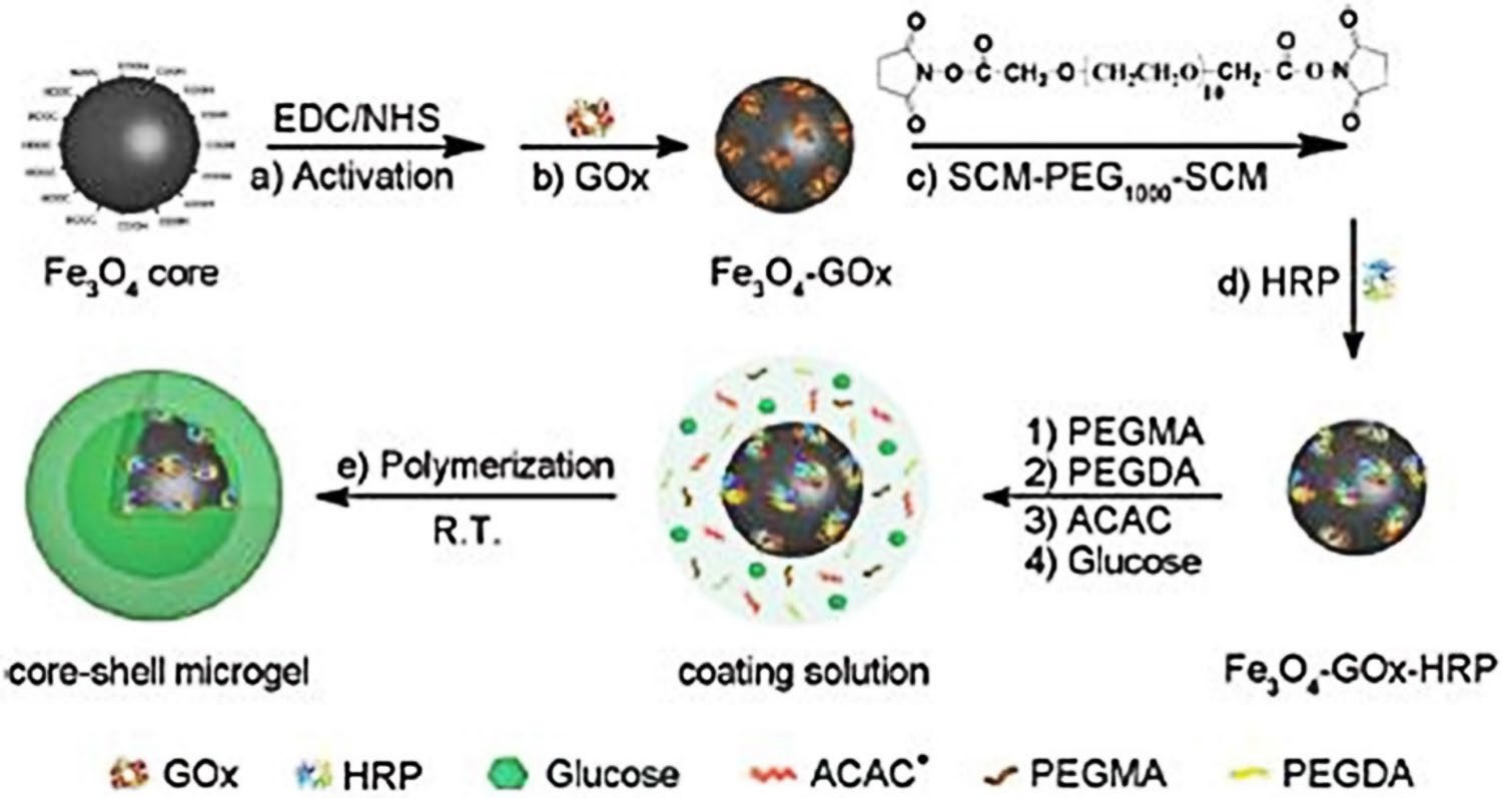
Synthesised magnetic core–shell microgels for single step colorimetric detection of glucose. Reprinted with permission from ref (Wu et al. 2015a) Copyright (2015) Royal Society of Chemistry

Recent developments in nanotechnology have also aimed towards the construction of novel enzyme mimetics (nanozymes) exhibiting biological oxidase, peroxidase, catalase, and superoxide dismutase-like activities (Gao et al. 2017; Karim et al. 2018; Lin et al. 2014d; Shah and Singh 2018). Nanozymes are nanomaterials possessing intrinsic biological enzyme-like properties, which offer several advantages over natural enzymes such as high stability and activity at varying conditions of pH and temperatures, cost effectivity, easy manipulation and multiple applications on a single platform. The shape, size, and composition controlled synthesis of nanomaterials provides easy modulation of their nanozymatic activity, which is one the major limitations with natural enzymes. Various metal and metal oxide-based nanomaterials such as IONPs, Gold, Silver, Copper, and nanosheets of graphene, MoS_2_, WS_2_ are shown to display horseradish peroxidase (HRP)-like activity. These enzyme-mimetic activities are shown to be used for the construction of non-enzymatic biosensors to test the levels/concentrations of glucose, glutathione, cholesterol, H_2_O_2_, urea, creatinine and biomarkers for cancer diagnosis (Gawande et al. 2016; Lin et al. 2014a, b; Mahato et al. 2018a, b; Vallabani et al. 2017; Wang et al. 2017; Zheng et al. 2011). These biomolecules are well-known biomarkers for several diseases, thus the developed biosensors could be used for the early diagnosis of these diseases. In general, the peroxidase-like activity of these nanozymes is observed at an optimum pH of 3–5, through the generation of hydroxyl radicals by Fenton reaction. For glucose detection, most of the colorimetric assays follow a two-step procedure. In the first step, glucose gets oxidized in presence of glucose oxidase (GOx) to release H_2_O_2_ at neutral pH. In the second step, the obtained H_2_O_2_ along with HRP or peroxidase mimetics are allowed to oxidize the substrates like 3,3′,5,5′-tetramethylbenzidine (TMB), 2,2′-azino-bis(3-ethylbenzothiazoline-6-sulphonic acid) (ABTS) and *O*-phenylenediamine (OPD) to form the colorimetric product at acidic pH. Although nanozymes degrade H_2_O_2_ rapidly and follow the oxidation of the products by hydroxyl radicals, still the reaction suffers from several shortcomings such as lower substrate affinity and specificity than natural enzymes. Such events lead to the lower performance of nanozymes, which ultimately faces limited applicability in sensing and biomedicine. Therefore, it becomes necessary to develop novel ways to enhance the catalytic activity of nanozymes. To address this issue, Yu et al. have studied the impact of citrate, glycine, polylysine, dextran and heparin coating on the peroxidase mimetic MNPs (Yu et al. 2009). They observed that the developed anionic NPs had high affinity towards TMB, whereas cationic NPs showed high affinity for ABTS. Based on the substrate affinity, they were able to detect glucose in the traditional two-step process and suggested that the nanozymatic activity can also be extended to other biomedical applications. Bhagat et al. have developed Gold-core/Cerium oxide-shell NPs exhibiting a multienzyme complex-like activity including catalase, SOD, and peroxidase enzyme-like activities. Kinetic parameter study related to peroxidase activity demonstrated that the core–shell nanozyme activity was comparable to the natural enzyme, horseradish peroxidase (HRP). In addition, authors also explained that oxidation of TMB was carried through electron transfer instead of hydroxyl radical participation. Additionally, the enzyme activity was conserved at a broad range of pH (2–11) and temperatures (up to 90 °C). However, the nanozyme exhibited optimum SOD and catalase activity at neutral pH and for peroxidase, it was found at acidic pH. Finally, the nanozymatic (peroxidase mimetic) activity was utilized for the detection of glucose in a range from 100 µM to 1 mM in a time span of 5 min and at pH 4 (Bhagat et al. 2017). Additionally, Wu et al. developed a magnetic core–shell microgel system with immobilized GOx and HRP molecules. Due to co-entrapment of enzymes, they utilized the magnetic microgel for the colorimetric detection of glucose in a single step at pH 5.5 (Fig. 3). Moreover, they suggested that this detection system can also be extended for detecting biomolecules through new oxidase or peroxidase platforms (Wu et al. 2015a).

**Fig. 4:**
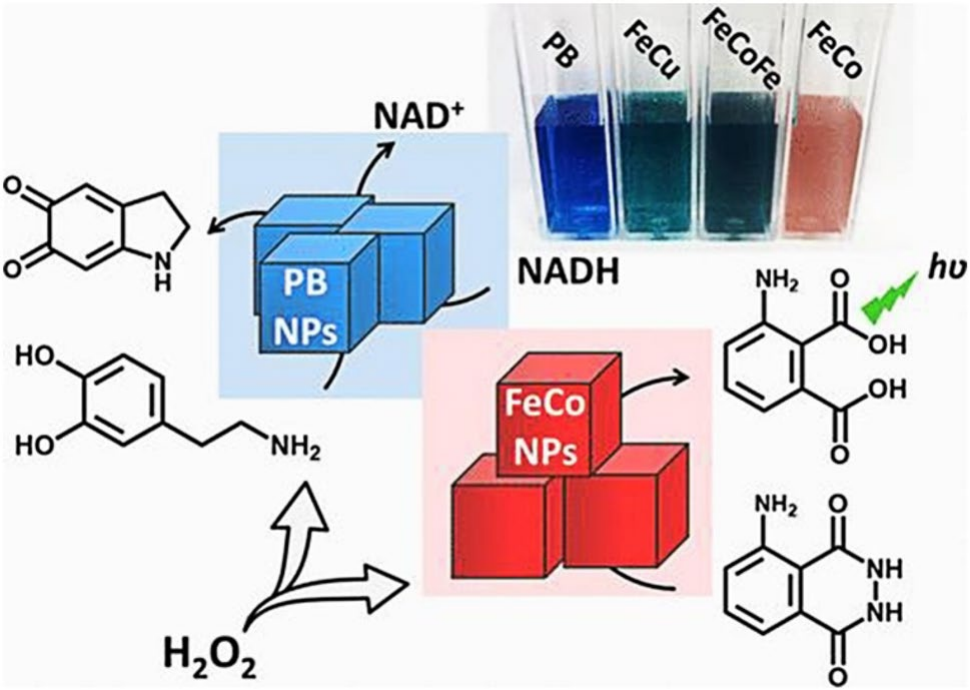
Prussian blue (PB), and the cyanometalate structural analogs, CuFe, FeCoFe, and FeCo, are examined as inorganic clusters that mimic the functions of peroxidases. Schematic showing PB NPs catalyzed oxidation of NADH by H_2_O_2_ to form NAD^+^ and chemiluminescence generation by the FeCo NPs catalyzed oxidation of luminol by H_2_O_2_. Reprinted with permission from ref (Vazquez-Gonzalez et al. 2017) Copyright (2017) American Chemical Society

**Fig. 5:**
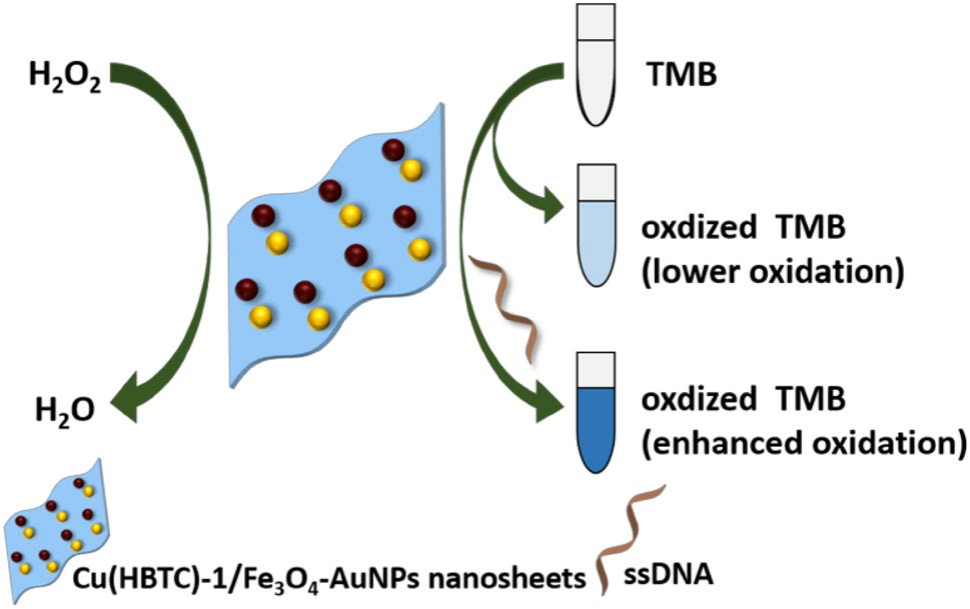
Schematic illustration of peroxidase-like activity and its controllability regulated by DNA of Cu(HBTC)–1/Fe3O4–AuNPs nanosheets. Reprinted with permission from ref (Tan et al. 2017) Copyright (2017) Royal Society of Chemistry

Composite materials have also been studied for the possible nanozymatic activities. In one such attempt, carbon nanotube/polyaniline-based metal oxide (Fe‚ Co‚ Ni) NPs are synthesized to show peroxidase mimetic activity using TMB and phenol/4-aminoantipyrine (4-AAP/phenol) to study the peroxidase-like activity and translated into a colorimetric method for glucose detection (Navvabeh Salarizadeh et al. 2017). Additionally, Vazquez-Gonzalez et al., have shown that NPs composed of the Prussian blue (PB), CuFe, FeCoFe, and FeCo inorganic clusters mimic the peroxidase activity. They suggested that PBNPs catalyze the oxidation of NADH by H_2_O_2_ to form NAD^+^ which can be applied for chemical transformations by NAD^+^ dependent enzymes such as ethanol dehydrogenase (Fig. 4). It was also found that the FeCo NPs catalyzed chemiluminescence generation in presence of H_2_O_2_ and luminol and extended this system for effective sensing of glucose (Vazquez-Gonzalez et al. 2017). Recently, bimetallic 2D nanosheets are also reported to act as biological peroxidase enzyme. In this context, Tan et al. synthesized bimetallic nanosheets exhibiting peroxidase-like activity, which can be modulated with the single-stranded DNA (ssDNA). The major attraction of this method was the switchability of peroxidase-like catalytic activity with DNA (Fig. 5). By modulating the enzyme-like activity of these nanosheets they achieved an ultra-sensitive detection of H_2_O_2_ with a range of 2.86–71.43 nM and comparable detection of glucose with a linear range of 12.86–257.14 µM (Tan et al. 2017).

### Iron oxide nanoparticles in electrochemical sensing and role of biomolecules in enhancing nanozyme activity

**Fig. 6:**
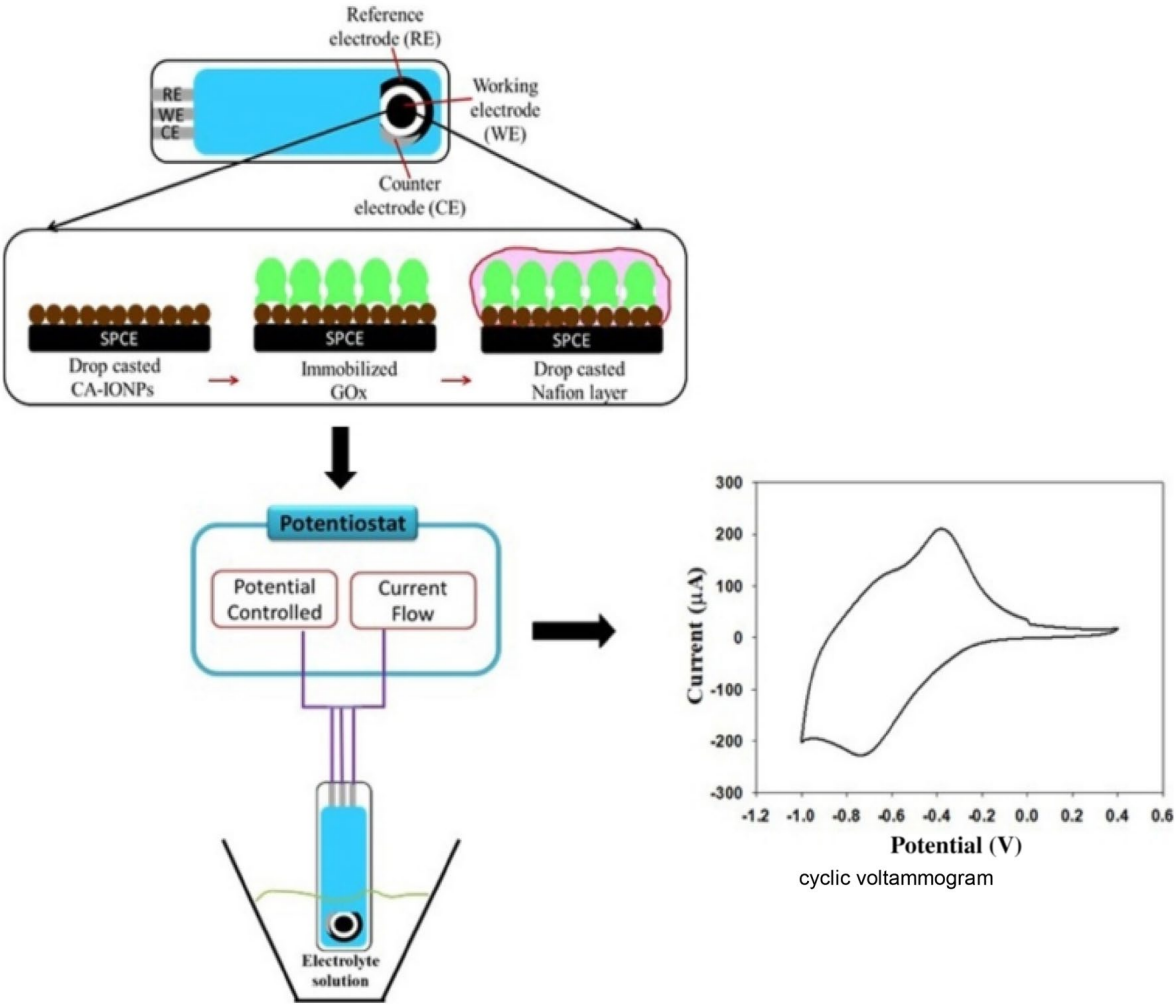
Schematic illustration of Nafion/GOx/IONPs/SPCE biosensor for electrochemical based detection of glucose. Reprinted with permission from ref (Nor et al. 2017) Copyright (2017) Elsevier

Unlike enzymatic reactions, nanomaterials exhibit the non-enzymatic way to detect the biomolecules. Since these methods are not constrained by the need of special conditions of pH and temperature, the process of detection does not get interference with other comparative biomolecules. Baby and Ramaprabhu et al. reported a superparamagnetic nanocomposite composed of SiO_2_ coated Fe_3_O_4_ NPs dispersed on multiwalled-carbon nano tubes (Fe_3_O_4_@SiO_2_/MWNT) exhibiting enhanced electron transfer ability and biocompatibility. This system was successfully applied to the development of a glucose and cholesterol sensor without any interference from other biomolecules and did not involve any enzyme (Baby and Ramaprabhu 2011). Further, Nor et al. developed a high sensitive biosensor for glucose detection using Nafion/GOx/IONPs/screen printed carbon electrode (SPCE). The amperometric biosensor exhibited a wide range of glucose detection with a lower limit of 7 µM (Nor et al. 2017) (Fig. 6). In the similar concept, Kacar et al. developed an amperometric biosensor for creatine monitoring using Fe_3_O_4_-nanoparticles-modified carbon paste electrodes. The method relies on two enzymatic catalyzed reactions, creatinase, and sarcosine oxidase to generate H_2_O_2_ and finally, the sensor sensitivity depends on the response towards H_2_O_2_ for creatine determination (Kacar et al. 2013). Since the creatine level in the human blood and urine acts as a clinical parameter to monitor muscle damage, this system could be of immense benefit to clinical monitoring of these biomolecules.

Composites of multiwalled-carbon nanotubes doped metal oxide NPs (NiO, ZnO, and Fe_3_O_4_) are coated on glassy carbon electrode and used for the sensitive detection of serotonin from body fluids such as urine. Serotonin is a well-known neurotransmitter and neuromodulator which plays a significant role in several biological processes like liver degeneration, endocrine regulation, anxiety, and depression. Hence, the determination of serotonin levels in the blood can assist to diagnose several diseases. Using this material, authors showed the voltammetry-based determination of serotonin, dopamine and ascorbic acid, simultaneously with high signal to noise ratio (Fayemi et al. 2017). Urea is another marker for kidney failure, obstructions in urinary tract, liver failure and other gastrointestinal problems. Ali et al. devised a potentiometric sensor by immobilizing urease enzyme over chitosan conjugated Fe_3_O_4_ NPs for detecting urea in the range of 0.1–80 mM (Ali et al. 2013). Andrea et al. developed IONPs for the sensitive and direct detection of biomolecules from biological samples. The NPs were functionalized with lipopolysaccharides obtained from a *Brucella* species and detected the presence of *Brucella* antibodies in serum. Further authors suggested that the method is versatile and NPs can be functionalized with different antigens on the surface, which could be used for easy identification of a variety of analytes from the body fluids (Fornara et al. 2008).

To increase the efficiency of nanozymes and overcome the pH constraints, synergistic molecules like nucleobases, nucleosides, nucleotides, and DNA are supplemented in the solution or coated on to the surface of NPs. This synergistic effect allows the design of simple and novel sensors for biomolecule detection at the desired pH (Lin et al. 2014c; Pu et al. 2017; Shah et al. 2015). Thus, to enhance the utility and translate the system into a point-of-care device platform, one-step method of detection is imperative. In this context, Vallabani et al. reported a novel strategy to overcome the limitation of acidic pH for hydroxyl radical generation and showed that with the use of ATP, IONPs exhibited excellent peroxidase mimetic activity at physiological pH. Moreover, the enzymatic activity was preserved over a wide range of pH and temperatures in presence of ATP. They utilized the property for single step detection of glucose at pH 7.4 and further extended to detect glucose level in human blood serum (Vallabani et al. 2017). Further to this study, Liang et al. synthesized novel NPs by growing coordinate polymer (CP) shell made of Fe^3+^ and Adenosine monophosphate (AMP) on Fe_3_O_4_ NPs. CP shell showed an advantage in encapsulating a wide variety of guest molecules like nucleic acids, proteins, fluorophores, and NPs. Authors explained that the shell has enhanced peroxidase-like activity due to Fe_3_O_4_ present in the core and applied this peroxidase nanozyme for glucose bio-sensing. GOx was entrapped in the shell of Fe_3_O_4_ NPs for glucose detection and monitored with a sensitivity of 1.4 µM (Liang et al. 2016). Additionally, Yang et al. reported that adenosine analogs with phosphate groups can enhance the peroxidase-like activity of Fe_3_O_4_ NPs. The improved activity was detected through the oxidation reaction of H_2_O_2_ and amplex ultra-red reagent generating fluorescence. Here the enhanced peroxidase activity of adenosine phosphate analogs showed the activity trend as AMP > ADP > ATP. Based on the protein adsorption on NPs authors also developed a turn-off system for detection of urinary proteins (Yang et al. 2017) (Table 2).

**Table 2:** Summary of bio-sensing applications shown by iron oxide nanoparticles

Nanoparticle/material	Size (nm)	Applications/results	References
Microgel embedded IONP-GOx-HRP	~ 200	Exhibited peroxidase like activity. Colorimetric detection of glucose was carried in a single step at pH 5.5	Wu et al. (2015a)
Prussian blue FeCo NPs	40–50	PBNPs catalyzed the oxidation of NADH by H_2_O_2_ to form NAD^+^ (showed dehydrogenase like activity). FeCo NPs catalyzed chemiluminescence generation in presence of H_2_O_2_ and luminol (showed Peroxidase like activity). Glucose detection was performed using FeCo NPs	Vazquez-Gonzalez et al. (2017)
Cu(HBTC)-1/Fe_3_O_4_-AuNPs nanosheets with ssDNA	5.99 ± 2.58	Enhanced TMB oxidation was observed in presence of single stranded DNA. 2D bimetallic immobilized MOF nanosheets were applied for detection of H_2_O_2_ (2.86–71.43 nM range) and glucose (12.86 to 257.14 µM range)	Tan et al. (2017)
Fe_3_O_4_@SiO_2_/MWNT (SiO_2_ coated Fe_3_O_4_ NPs dispersed on Multiwalled-carbon nano tubes)	5–15	Biosensor was applied for detection of glucose (3 µM–14 mM range) and cholesterol (10 µM–4 mM range)	Ramaprabhu (2011)
FeNPs@Co_3_O_4_ (IONPs loaded in Co_3_O_4_ hollow nanocages)	900	Applied for glucose detection with a linear range of 0.5–30 µM (limit of detection was 0.05 µM)	Zhao (2018)
Fe_3_O_4_-nanoparticles-modified carbon paste electrodes	–	Creatinine was determined with a detection limit of 2.0 × 10^−7^ mol L^−1^	Kacar et al. (2013)
Graphene oxide/Fe_3_O_4_ nanocomposite	50	Biosensor for determination of glucose, with a range of 0.5–10 mM	Wang (2018)
MWCNT doped with Ni, Zn, Fe	10–50	Serotonin was determined with a detection limit of 5.98 × 10^−3^ µM–62.8 µM	Fayemi et al. (2017)
Chitosan-IONPs with urease	–	Applicable for the detection of urea	Ali (2013)
IONPs	19.5	*Brucella* antibodies detection with a detection limit of 0.05 µg mL^−1^	Fornara et al. (2008)
Fe_3_O_4_ NPs	13 ± 3.5	ATP-mediated peroxidase like activity of Fe_3_O_4_ NPs was observed at pH 7.4. Glucose detection was carried in a single step at physiological pH with a colorimetric detection limit of 50 µM	Vallabani et al. (2017)
Fe_3_O_4_ NPs with Fe^3+^ AMP shell	10–20	Glucose detection was demonstrated with a detection limit of 1.4 µM	Liang et al. (2016)
Fe_3_O_4_ NPs	~ 13	Exhibited peroxidase like activity. Can be applicable as a fluorescent turn-off system for urinary protein detection	Yang et al. (2017)

## Iron oxide nanoparticles in hyperthermia and photo thermal therapy

**Fig. 7:**
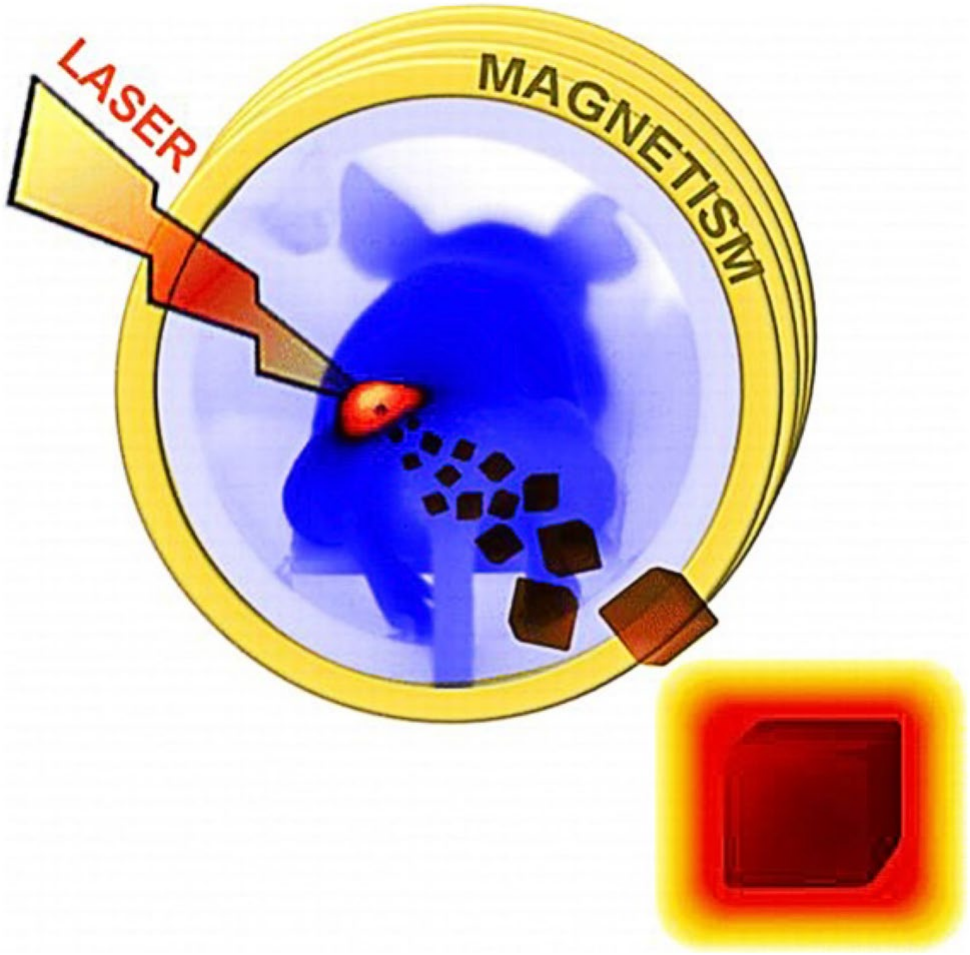
Dual mode magneto-photo-thermal approach using iron oxide nanocubes for tumor ablation. Reprinted with permission from ref (Espinosa et al. 2016) Copyright (2016) American Chemical Society

Hyperthermia is a thermal therapy to produce heat near a local or a systemic tumor by energy sources like microwaves, radio waves, ultrasound energy and magnetism. Recently, it has been realized that the conventional methods of cancer treatment suffer from several limitations such as side effects, drug resistance, low availability of drug at the site of action, fast renal clearance, etc. These challenges have allowed researchers to combine the chemotherapy and radiotherapy with hyperthermia. In magnetic hyperthermia, the intratumorally injected IONPs generate heating effect after exposing to an external magnetic field and induce the cell death near the tumor zone (Elham Cheraghipour 2012; Kolosnjaj-Tabi and Wilhelm 2017). Due to poor cellular architecture, cancer cells are very prone to be damaged by the slight increase in the surrounding temperature. Additionally, using hyperthermia strategy, the temperature of the surrounding environment can be increased up to 55–60 °C, which can be very well withstood by normal healthy cells, but not by cancerous cells. Moreover, IONPs can be used to prepare synergistic nano-hybrids tuned for magnetic hyperthermia and photothermia. Photothermal therapy is also possible with the use of other nanoparticles such as anisotropic nanostructures of gold, copper, silver, carbon nanotubes, and graphene NPs (Boca et al. 2011; He et al. 2014; Liu et al. 2007; Yavuz et al. 2009). Other morphologies of IONPs are also developed for the photothermal treatment of diseases. Espinosa et al. have used nanocubes of IONPs and showed that when these nanocubes are exposed to the magnetic field as well as near-infrared laser irradiation, two- to five fold amplification in the production of heat was generated than when used magnetic field alone (Fig. 7). Moreover, it was demonstrated the dual-mode stimulation generated efficient heat with low iron concentration (0.25 M) and acceptable laser power irradiation (0.3 W/cm^2^) and resulted in a complete cell death and solid tumor suppression (Espinosa et al. 2016). In another attempt, Niu et al. developed a nanosystem comprising of IONPs (Fe_3_O_4_), indocyanine green (ICG) and perfluoropentane (PFP) encapsulated in poly (lactideco-glycolide) (PLGA) nanoparticles for NIR induced PTT. The experiments with MCF-7 tumors in mice showed that these multifunctional NPs can enhance tumor ablation upon NIR laser irradiation and can act as a key photothermal agent against the tumors (Niu et al. 2017).

**Fig. 8:**
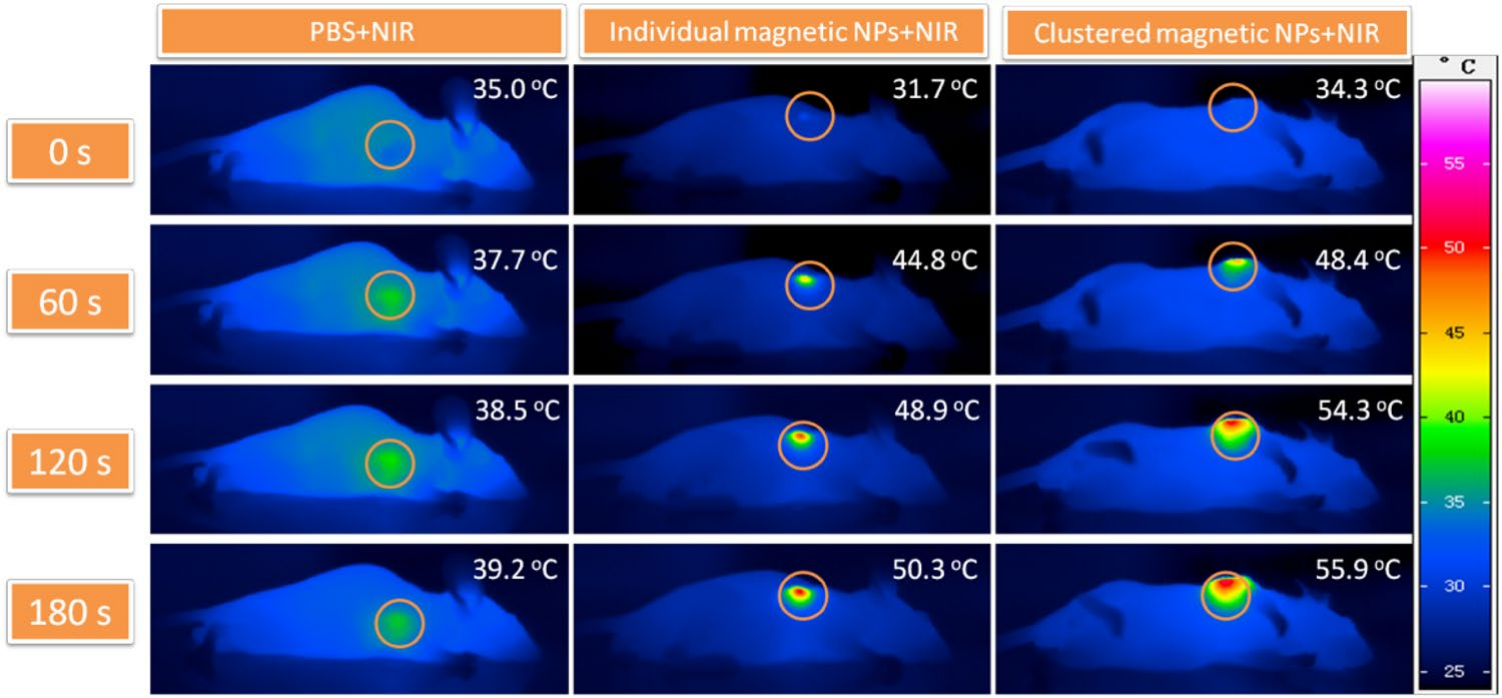
Infrared thermal images of phosphate buffered saline (PBS), individual and clustered magnetic Fe_3_O_4_ NPs with the concentration of 100 µg/mL injected in A549 tumor sample under NIR laser irradiation for 0–180 s. Reprinted with permission from ref (Shen et al. 2015) Copyright (2015) Elsevier

Addressing hyperthermia, Mazario et al. synthesized 10 nm sized MNPs functionalized with HAS protein and the experimental data revealed these functional materials have the potential role in mediating magnetic hyperthermia by enhancing the temperature of cancer cells. Moreover, authors suggested that heating could induce irreversible damage to cellular proteins and enzymes and thereby the regulation of apoptosis in cells and tissues (Mazario 2017). In a related study, Shen et al. synthesized a magnetic nanocluster for PTT using near-infrared light irradiation. They found that after radiation exposure clusters of Fe_3_O_4_ produced more heating thereby impart significant cytotoxic to A549 cells (human lung carcinoma) compared to bigger sized Fe_3_O_4_ NPs. The mechanistic studies revealed the cell death was caused due to apoptosis, but not necrosis. Further, in vivo studies demonstrated that these clusters can be applied for promising tumor treatment through PTT (Fig. 8) (Shen et al. 2015). Chen et al. have developed highly crystalline IONPs (HCIONPs) coated with the anti-biofouling polymer and used then for photothermal cancer therapy. Results revealed NPs were effectively accumulated in the tumor site of SUM-159 tumor-bearing mouse though permeability and retention effect. Further laser irradiation exhibited complete tumor regression within 3 weeks compared to control. Authors suggested that enhanced PTT was due to high crystalline and preferred lattice plane orientations of as prepared HCIONPs compared to normal Fe_3_O_4_ NPs (Hongwei Chen 2014).

**Fig. 9:**
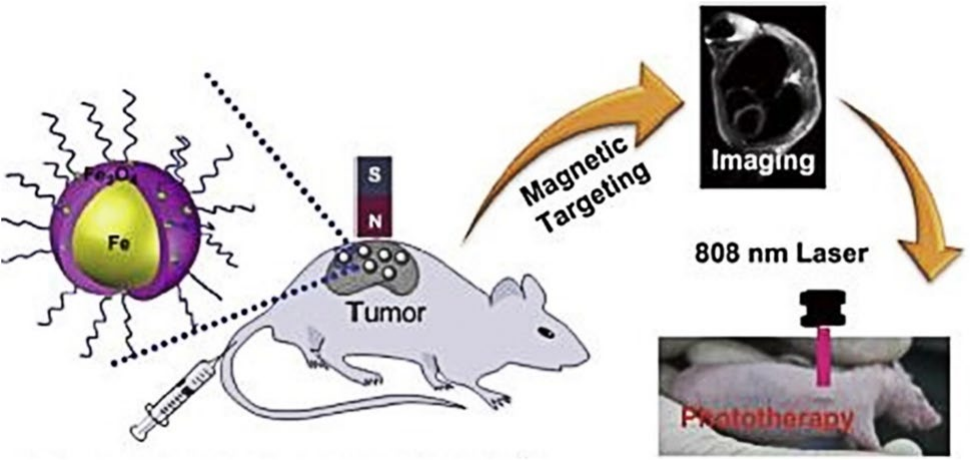
Schematic illustration of magnetic targeting, MRI and NIR photothermal therapy by multifunctional PEGylated Fe/Fe_3_O_4_ NPs. Reprinted with permission from ref (Zhou et al. 2014) Copyright (2014) Elsevier

Multifunctional IONPs are also designed and have shown better results than their uni-functional counterparts. In this context, PEGylated Fe/Fe_3_O_4_ NPs have been developed for exhibiting triple functions comprising PTT, targeting, and MRI (Fig. 9). Targeting of cells was achieved with neodymium magnet placed beside a xenograft tumor developed from HeLa cells. The multi-modality allowed the NPs to accumulate in the tumor region, therefore, exhibited intense MRI signal with high photothermal activity (Zhou et al. 2014). PEG coating on nanoparticle surface imparts biocompatibility and avoidance to the reticuloendothelial system (RES), which renders the long-term circulation in blood plasma (Larson et al. 2012). Therefore, biocompatible nanomaterials would be an ideal candidate for multifunctional applications cell targeting, MRI, sensing, hyperthermia, and PTT. For instance, Shen et al. synthesized a carboxymethyl chitosan coated Fe_3_O_4_ NPs, which exhibited extremely low toxicity and high PTT efficiency. Furthermore, they stated that these NPs platform can be easily fabricated for multiple applications (Shen et al. 2013) (Table 3).

**Table 3:** Summary of iron oxide based nanoparticles for hyperthermia and photo thermal therapy

Nanoparticle/material	Size (nm)	Applications/results	References
IONPs (nano-cubes)	20	The dual mode (hyperthermia and PTT) of treatment amplified the heating effect by two- to fivefold in comparison with magnetic stimulation alone. Results showed that in both in vitro (SKOV3) (ovarian cancer), PC3 (prostate cancer) and A431 (epidermoid cancer) and in vivo (A431 cancerous cells were injected in nude NMRI mice) complete cell death was observed after dual mode exposure	Espinosa et al. (2016)
ICG/Fe_3_O_4_ loaded PLGA NPs	Fe_3_O_4_: 10 Total shell: ~300	Used as an efficient treatment by PTT. In vitro treatment of NPs to MCF-7 breast cancer cells confirmed the damage to cells and in vivo studies demonstrated IONPs can be used as an effective agent for tumor ablation	Niu et al. (2017)
Carboxyl-amine functionalized SPIONs based ferrofluids	~ 20	In vitro hyperthermia studies revealed terephthalic acid (TA) and aminoterephthalic acid (ATA) coated SPIONs induced ~ 90% cell death in breast cancer cells (MCF-7)	Kandasamy (2018a)
IONPs with HSA	10	Used for thermal therapy. MNPs exhibited a saturation magnetization of 63 emu g^−1^ at 310 K and produced a localized heat in presence of an alternating magnetic field	Mazario (2017)
Clustered magnetic Fe_3_O_4_ NPs	Fe_3_O_4_: 15 Clustered Fe_3_O_4_: 225	Used for PTT. The clustered NPs induced high temperature and proved to be more cytotoxic against A549 cells both in vitro and in vivo	Shen et al. (2015)
SPIONs	6–10	Hyperthermia based thermotherapy for liver cancer treatment	Kandasamy (2018b)
Crystallized IONPs (HCIONPs)	15	Showed effective PTT against SUM-159 tumor-bearing mice	Hongwei Chen (2014)
PEGylated Fe@ Fe_3_O_4_ (PEGylated iron/iron oxide core/shell NPs)	13.4 ± 0.8	These multifunctional NPs can be applied for targeting, MRI imaging and PTT	Zhou et al. (2014)
Fe_3_O_4_@CMCT (carboxymethyl chitosan stabilized Fe_3_O_4_ NPs)	177	Used for PTT. NPs were found accumulated in the mice tumor region and PTT induced the increase in temperature up to ~ 52 °C	Shen et al. (2013)

## Iron oxide nanoparticles as delivery agents

### Drug delivery applications

Effective treatment of cancer still remains a major challenge in medicine due to several problems such as drug/multidrug resistance, lack of selective targets for a tumor (El-Boubbou 2018; Vasir and Labhasetwar 2005). Nanotechnology has recently shown some success in effective cancer treatment due to the unusual properties of materials. Among several NP types, IONPs have gained the most attention for applications in nanomedicines due to some of the key attributes, including stable colloidal suspension, resistance to in vivo degradation, presence of large surface area to graft targeting moieties, high payload delivery of drugs, synergistic activity in improving the sensitivity of drugs towards cancer treatment, and reversing the resistance of cancerous cells (Bahrami et al. 2017; Ulbrich et al. 2016).

Traditional methods of cancer treatment include surgery, chemotherapy, radiotherapy, and combinational therapy to alleviate the tumors. However, damage to surrounding cells around a tumor and radio-resistance of cells limits the effectiveness of these conventional therapies. The multifunctional nature of IONPs has been recently exploited for the effective drug delivery for cancer and other disease treatment. Pirayesh Islamian et al. used superparamagnetic mesoporous hydroxyapatite conjugated doxorubicin and deoxy-D-glucose nanocomposites to boost breast cancer chemo and radiotherapy (Pirayesh Islamian et al. 2017). They worked on SKBR3 and T47D breast cancer cell culture models and reported that the cell viability was significantly decreased with combined nanocomposite effect compared to the radiotherapy alone. Mechanistically, they found that targeting of breast cancer cell was achieved with deoxy-d-glucose moiety conjugated on IONPs surface and doxorubicin acted as therapeutic agent, thus the combined effect leads to the improved breast cancer radiotherapy by increased localization of NPs. When investigated further, it was found that the extent of cytotoxicity in tumor cells was much more, with minimum side effects and damage to normal healthy cells. In another strategy, Ye et al suggested that Fe_3_O_4_ NPs can increase the efficacy of cryoablation; a process uses extreme cold conditions to treat cancerous cells. Their data indicated that Fe_3_O_4_ NPs altered intracellular ice formation ability during freezing, recrystallization, and thawing, which leads to the enhanced killing of MCF-7 cells. Therefore, the idea of enhanced ablation using IONPs can be successfully applied to effectively treat tumors in near future (Ye et al. 2017).

**Fig. 10:**
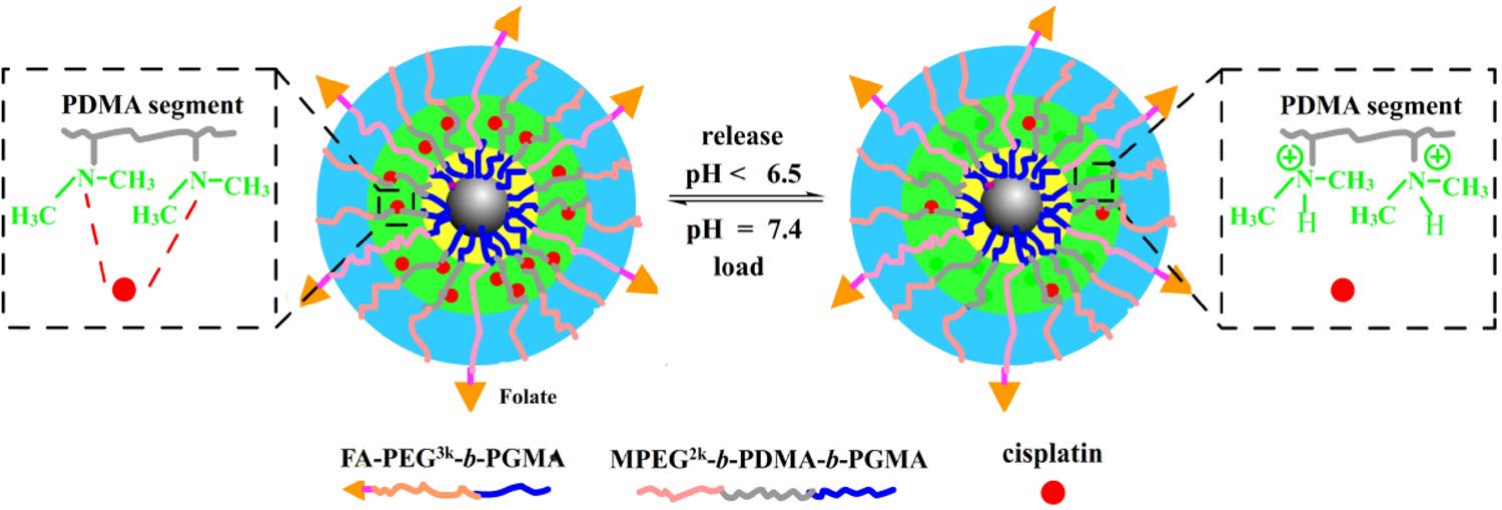
Schematic representation of the structure of FA-CIS-POLYMER-Fe_3_O_4_ nanoparticles and cisplatin loading and release. Reprinted with permission from ref (Yan Zhang 2014) Copyright (2014) Springer Nature

Cisplatin is platinum-based anticancer agent reported to treat various types of cancers including lung, testicular, bladder, ovarian, breast, and brain tumors (Dasari and Tchounwou 2014). However, the excess usage of cisplatin is also reported to exhibit various side effects such as kidney damage, neurotoxicity, bone marrow suppression, heart diseases, and allergic reactions (Barabas et al. 2008). It is also well known that the drug resistance shown by tumor cells limits their probability of clinical trial success. To overcome these limitations, platinum drugs are suggested to be encapsulated into polymers or loaded into multifunctional nanocomposites for effective treatment of cancers. In this context, Yan Zhang et al. fabricated a nanocomposite comprising of Fe_3_O_4_ core and polymeric inner shell covered with PEG, and folate groups, and the cisplatin was encapsulated into the inner shell through coordination of amino groups (Yan Zhang 2014). The in vitro release kinetics of cisplatin showed better release at acidic pH (pH ~ 4.5) and resulted in a cytotoxic response in HeLa cells through ligand-mediated targeting of folate receptors (overexpressed on HeLa cells) (Fig. 10). Additionally, Ebrahimi et al. have developed a PLGA-PEG copolymer system by emulsion method, which encapsulated Fe_3_O_4_ NPs with doxorubicin (DOX) drug. With the controlled DOX release in tumor cells, this biodegradable nanocomposite was designed to minimize the drug uptake in normal cells and also to control the drug amount and targeting via copolymer coated IONPs and pH. The results revealed that the drug release was high at acidic pH and found effective as a chemopreventive and chemotherapeutic agent for effective treatment of lung and other solid tumors (Ebrahimi 2014a, b).

**Fig. 11:**
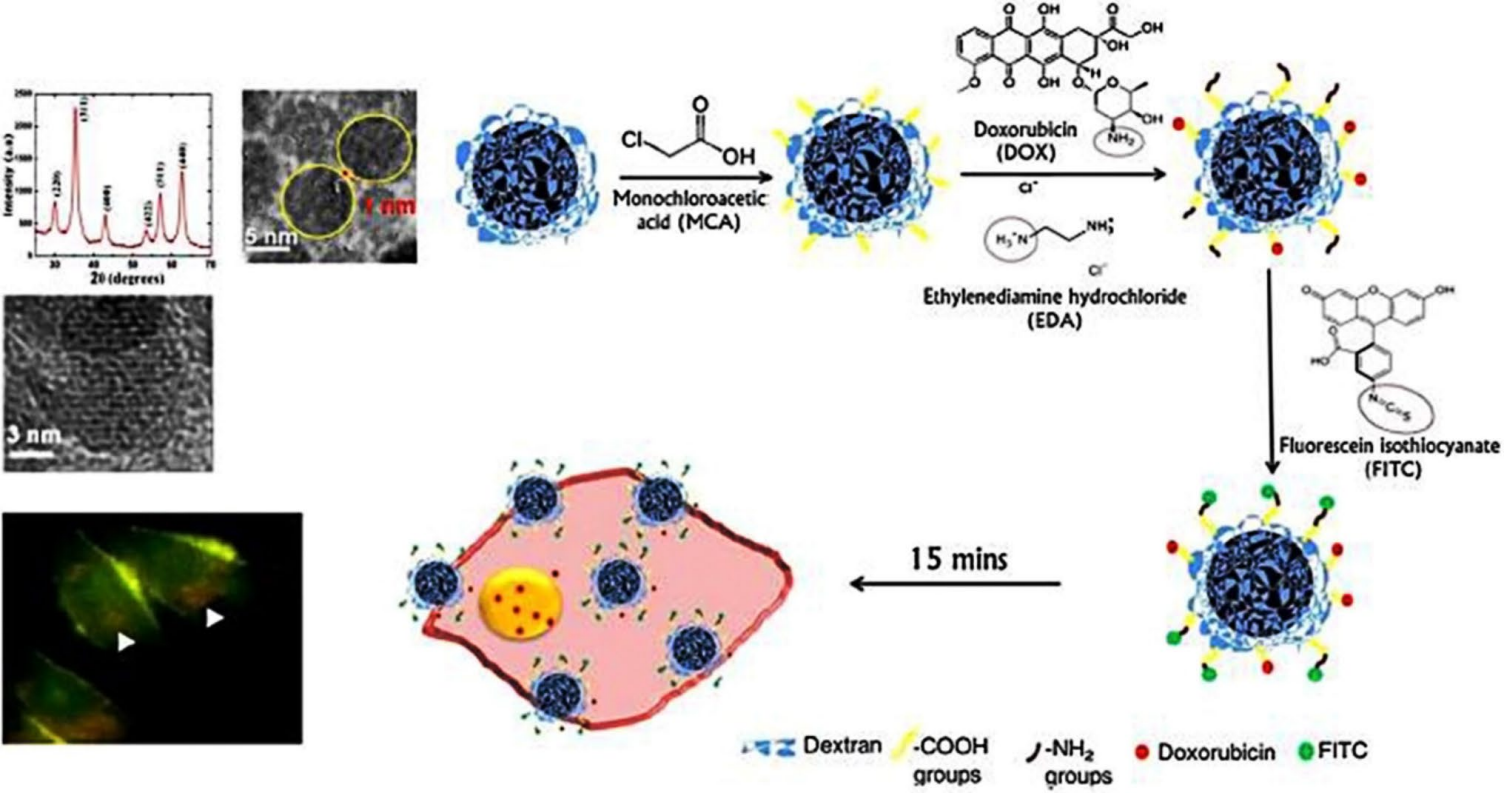
Synthesis and functionalization of superparamagnetic iron oxide (SPIO) nanoparticles for rapid cellular entry and release of the cancer drug Doxorubicin (DOX) in human pancreatic cancer cells. Dextran coated Fe_3_O_4_ core with DOX (red fluorescence) and FITC (green fluorescence) surface conjugation chemistry, and the rapid entry (15 min) and intracellular release and accumulation of the cancer drug in the nucleus (white arrow head) of human pancreatic cancer Mia Paca-2 cells.. Reprinted with permission from ref (Arachchige et al. 2017) Copyright (2017) Elsevier

Epidermal growth factor receptor (EGFR) is also a well-known targeting moiety, which has been well-explored for the targeted drug delivery in cancer cells. Utilizing this strategy, Xupeng et al. have developed a multifunctional nanocomposite consisting of IONPs and shown applications in the diagnosis, targeting, and chemo and photothermal therapy of cancer (Xupeng Mu 2017). The nanocomposite was designed by conjugating EGFR antibody on polydopamine-coated Fe_3_O_4_ NPs and loaded with DOX. This nanocomposite exhibited pH and NIR triggered drug release, which resulted in effective inhibition of colon cancer cells (DLD-1, exhibiting overexpression of EGFR) due to synergistic chemo and photothermal therapy. Additionally, these nanocomposites were also utilized for T_2_ contrast generation to follow the tumor growth by MRI scanning under in vivo experimental condition. In another attempt, Arachchige et al., synthesized dextran coated IONPs and conjugated them with DOX and fluorescein isothiocyanate (Fig. 11). The so produced IONPs laid an efficient combination platform for imaging through Fe_3_O_4_ contrast (MRI), red fluorescence (auto fluorescing DOX) and green fluorescence (fluorescein isothiocyanate). Results revealed that significant drug internalization was obtained in pancreatic cancer cells (MIA PaCa-2 cells). Further, the dual-fluorescent tracking mode showed that the DOX was cleaved from the NPs and accumulated at the targeting site (nucleus). Such strategies could also be utilized for conjugation of an additional antibody to the existing NPs platform, which could be helpful in a tumor specific therapy (Arachchige et al. 2017).

Jia et al. constructed a nanocarrier with PLGA polymer encapsulating IONPs and DOX. The drug internalization study was performed in multiple cancer cell culture models such as Lewis lung carcinoma (LLC), human osteosarcoma (OS-732), and murine-leukemic monocyte–macrophage (RAW 264.7) cell lines and results revealed that IONPs-DOX combination was internalized in cells in higher amount with respect to when DOX alone was used. In addition, a higher concentration of IONPs-DOX internalization induced apoptosis in LLC cell line. Under In vivo experiments, results showed that more anti-tumor effect was seen with combination therapy, which was further improved in presence of external magnet (Jia et al. 2012).

The blood–brain barrier is a semipermeable membrane that protects neural tissue from toxins and exogenous substances. The tight junctions in the endothelial cells around the capillaries are responsible for the barrier formation. Daunorubicin (DNR) is a potent topoisomerase inhibitor and used as an effective chemotherapeutic agent to treat leukemia and neuroblastoma. Xuhua et al. synthesized DNR loaded Fe_3_O_4_ NPs and found the formulation was efficient enough to cross the blood–brain barrier which separates the circulating blood from the brain. Results suggested that the combination therapy can trigger apoptosis in glioma cells and thus used as a promising agent to cross the otherwise impermeable blood–brain barrier and treat brain tumors (Xuhua Mao 2016). Experimental data indicated that the formulation increased the barrier permeability through opening tight junctions via controlling the expression of cell–cell adhesion proteins (E-cadherin, ZO-1, and Claudin-1). Homoharringtonine (HTT) conjugated Fe_3_O_4_ NPs were synthesized by Chen et al. to inhibit leukemia. Results showed NPs and drug combination enhanced the inhibitory effect on myeloid leukemia cell lines compared to HTT exposure alone. They found cell death was caused due to apoptosis and observed cell cycle arrest at G0/G1 phase. Moreover, in vivo studies explained the combination worked efficiently to decrease the tumor volume compared to drug alone (Chen et al. 2016). A similar study explained the combination of Fe_3_O_4_ with adriamycin or daunorubicin drugs showed a potential inhibitory effect on lymphoma. A detailed study on Raji cells explained that the inhibition was due to induction of apoptosis which was further enhanced in the presence of NPs (Hongmei Jing 2010). Huilan et al. designed a nanocomposite encompassing Fe_3_O_4_ NPs and an anticancer drug ursolic methyl ester. They demonstrated combination treatment increased the rate of apoptosis in drug-resistant human leukemia cells (KA cells) compared to the drug alone. The authors suggested that this approach to inhibit the growth of drug-resistant cancer cells can be used as an alternative method to treat other cancers as well (Huilan Yue 2016).

Osteoporosis is a condition where bones become weak and brittle due to an imbalance between osteoblasts and osteoclasts (OCs). Thus, reducing the OCs activity, which involves the breakdown of bone tissues, can improve the bone damage. To overcome the effect a composite nanosystem was developed using Fe_3_O_4_ NPs with alendronate drug for treating osteoporosis. In this novel strategy, after the delivery of NPs near osteoclasts (OCs) cells, a dose of radiofrequency was applied to induce thermolysis to OCs. It was also suggested that these multifunctional NPs with their drug delivery, thermolysis, and contrast (MRI) generation ability can be used as a potential therapeutic and imaging agent for osteoporosis (Lee et al. 2016).

### Gene delivery applications

**Fig. 12:**
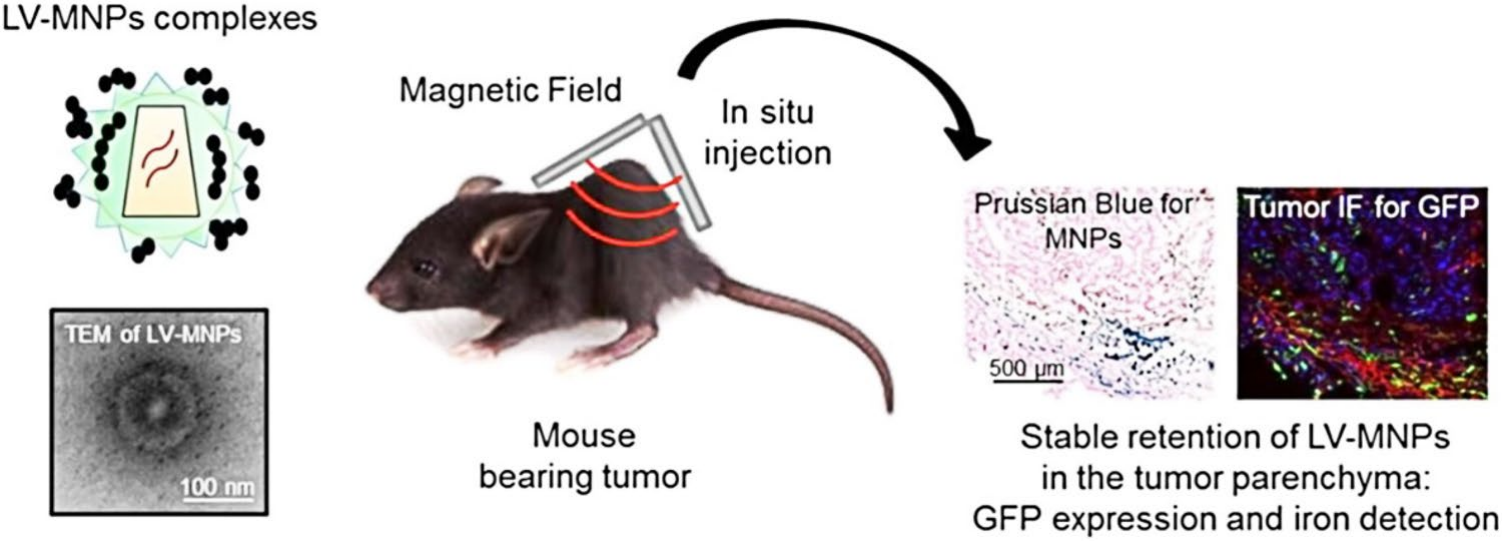
Magnetic nanoparticles (MNPs) coupled with lentiviral vectors (LVs) as multifunctional and efficient tools to selectively induce transgene expression in solid tumor for therapeutic purposes. Reprinted with permission from ref (Borroni et al. 2017) Copyright (2017) Elsevier

Gene delivery is a therapeutic technique for the delivery of nucleic acids instead of drugs or surgery to treat diseases. In general, it is a great challenge to deliver biological drugs like siRNA, and plasmid DNA to target cells or tissues without being damaged by nucleases. Therefore, to overwhelm the damage, nanocarriers like IONPs have been used to transport the genes and release at the intended target sites (Kievit and Zhang 2011). Borroni et al. developed a novel vector that can deliver therapeutic genes for treating tumors. The gene-carrier contained IONPs conjugated with lentiviral vectors to deliver target gene at the area of interest (Fig. 12). To check the efficiency, this nanocarrier was injected with a reporter GFP gene (green fluorescent protein) into a tumor bearing mice and found sustained gene expression near the target areas. Their data suggest that in future LV-MNPs can be successfully tailored to deliver therapeutic genes for selectively inhibiting the growth of tumors (Borroni et al. 2017). Additionally, Mahajan et al. designed a novel IONPs carrier to stop the progression of pancreatic cancer. IONPs were coupled with siRNA (siPLK1) directed against Polo-like kinase-1 (cell cycle-specific serine-threonine-kinase). Moreover, siPLK1-IONPs were conjugated to membrane translocation peptide (myristoylated polyarginine peptides (MPAP)) for driving endosomal escape and mediating transport to the cytoplasm and a tumor-selective peptide underglycosylated MUC1 (uMUC1)-specific peptide (EPPT1) to increase intracellular and tumor-specific delivery. The experimental data revealed the significant accumulation of NPs and resulted in efficient PLK1 silencing that leads to tumor suppression through increased apoptosis (Mahajan et al. 2016).

Cheong et al. synthesized IONPs-based carrier loaded with water-soluble chitosan and linoleic acid (SCLNs) for targeting hepatocytes. Authors confirmed the NPs localization in the liver cells by injecting a nuclear isomer (Technetium-99m) labeled SCLNs in mice using nuclear and magnetic resonance imaging. Further SCLN/enhanced GFP (pEGFP) complexes transfected in primary hepatocytes, intravenous administration in mice showed a significant increase in GFP expression. In addition, the gene silencing was effectively achieved by injecting SCLN complexes containing specific siRNA into mice. Thus, the results suggested that SCLNs can be used as a useful platform for imaging and gene delivery simultaneously (Cheong et al. 2009).

In addition, Ling-Feng et al. evaluated the use of IONPs as ideal gene-carrier for PTEN (Phosphatase and tensin homolog) gene delivery to reverse cisplatin-resistance in lung cancer. PTEN acts as a tumor suppressor gene and its inactivity causes the development of several cancers. A549/CDDP (cisplatin-resistant) cells were transfected with wild PTEN gene expression plasmid (pGFP-PTEN) using IONPs and liposomes as carriers. It was observed The IONPs mediated PTEN transfection showed higher efficiency and more PTEN expression compared to empty liposome-mediated transfection. Further, PTEN transfection increased the apoptotic cell population and enhanced the sensitivity of A549/CDDP cells to cisplatin indicating PTEN can be an effective target against cisplatin-resistant lung cancer cells (Ling-feng Min 2012) (Table 4).

**Table 4:** Summary of iron oxide NPs applications in drug delivery and gene delivery

Nanoparticle/material	Size (nm)	Applications/results	References
IONPs coated with 2-deoxy-d-glucose and DOX	Pore size: 12	NPs enhanced chemo-radiotherapy efficiency in breast cancer cells through targeting. Results showed the combined NPs treatment with doxorubicin and 2-deoxy-d-glucose boosted the breast cancer cure through improved radiotherapy	Pirayesh Islamian et al. (2017)
Fe_3_O_4_ NPs	9	Used for treating tumors through cryoablation therapy. Results indicated MCF7 cells were killed efficiently by cryoablation	Ye et al. (2017)
Fe_3_O_4_ NPs @PEG, folate and cisplatin (Folic acid-Polymer conjugated Fe_3_O_4_ NP_s_ with cisplatin)	~ 10	Used for ligand-mediated targeting and chemotherapy. Cisplatin-loaded NPs showed concentration dependent cytotoxicity in HeLa cells. Moreover folate conjugation exhibited more cytotoxicity compared to non-conjugated NPs	Yan Zhang (2014)
DOX encapsulated Fe_3_O_4_	~ 12	Used as a nano-carrier for anticancer drugs like doxorubicin. NPs can be applied as a chemotherapeutic system for treating lung cancers	Ebrahimi et al. (2014a, b)
Polydopamine coated Fe_3_O_4_ with EGFR antibody and DOX	~ 60	Used as a multifunctional composites in diagnosis (MRI imaging) and cancer treatment (chemo-photo thermal therapy). Results showed the combination therapy is efficient enough in killing EGFR expressed tumor cells (colon cancer)	Xupeng Mu (2017)
Dextran coated IONPs with FITC and DOX	~ 8	Used for drug delivery with multimodal imaging (MRI and FITC Fluorescence) and cancer treatment (drug and hyperthermia) Can be applied for treating pancreatic cancers	Arachchige et al. (2017)
MNPs encapsulated in PLGA with DOX	MNPs: 4–6 DOX-MNPs: 200–300	Used as a nano-carrier for drugs like doxorubicin. Results showed DOX-MNPs were internalized in to lung cancer cells (Lewis lung carcinoma cells) and induced apoptosis. Moreover in vivo studies revealed more anti-tumor activity in presence of an external magnetic field	Jia et al. (2012)
DOX loaded Fe_3_O_4_-reduced graphene oxide	8–10	Showed maximum inhibition of HeLa cells with hyperthermia assisted treatment	Gupta (2018)
Daunorubicin loaded Fe_3_O_4_ NPs	100	Used to treat brain glioma. Results showed this drug loaded NPs can be efficiently delivered into blood brain barrier and can act as promising drug to treat blood tumors	Xuhua Mao (2016)
Homoharringtonine conjugated Fe_3_O_4_ NPs	11.2	Used for in vitro and in vivo chemotherapy towards hematological malignancy. Results indicated drug conjugated MNPs injected in tumor bearing mice (leukemia) showed a significant decrease in tumor growth compared to drug treatment alone	Chen et al. (2016)
Liposome with paclitaxel and SPIONs	159–168	Comprising both MRI and antitumor characteristics. Results showed the tumor growth was supressed in MDA-MB-231 tumor-bearing mice compared to controls	Zheng et al. (2018)
Fe_3_O_4_ NPs with adriamycin and daunorubicin	–	Used as a combination therapy to treat lymphoma. Results revealed increased apoptosis in Raji cells and upregulation of p53, down regulation of NF-kB was observed with NPs drug combination treatment	Hongmei Jing (2010)
Cetuximab-IONPs	–	Both in vitro and in vivo studies revealed anti-tumor efficiency against gliomas	Freeman et al. (2018)
Fe_3_O_4_ NPs with urosilic metyl esters	10	Used as anti-cancer agent for leukaemia. NPs and the drug combination induced apoptosis in drug-resistant human leukemia KA cells	Huilan Yue (2016)
Fe_3_O_4_ NPs with alendronate	~ 20	Used for treating osteoporosis. Results showed NPs-drug exposure decreased the survival rate of osteoclasts compared to control cells and osteoblasts	Lee et al. (2016)
LV-MNPs	10–20	Can be applied as a combined therapeutic system to target gene expression in cancer cells	Borroni et al. (2017)
IONPs with siRNA	IONP core: 9.81 ± 3.73 Conjugated IONs: 12.3 ± 1.4	Used for treating pancreatic cancers. The nano-conjugate with siRNA resulted in efficient PLK1 silencing and halted the tumour growth with increase in apoptosis	Mahajan et al. (2016)
IONPs loaded chitosan–linoleic acid NPs	12	Used as a gene delivering system for targeting hepatocytes and gene silencing	Cheong et al. (2009)
IONPs with PTEN gene	–	Used as gene carriers for PTEN and applied for reversing cisplatin-resistance in lung cancer	Ling-feng Min (2012)

## Iron oxide nanoparticles as broad spectrum antimicrobial agent

Although IONPs have shown tremendous applications in drug delivery, phototherapy, and chemotherapy, they have also found overarching potential as the antimicrobial and antifungal agent. In the context of drug resistance in microbes, the developments of new drugs or novel strategies for efficient destruction of these pathogens need immediate attention. It is well known that antimicrobial resistance poses a catastrophic threat to humans because if it continues to grow with the current pace for 20 more years, it is estimated that people visiting hospitals for even minor surgery may die due to an ordinary microbial infection, which cannot be treated by antibiotics. It is estimated that the number of deaths due to microbial infection would surpass the mortality due to cancer or diabetes in few decades. Therefore, novel antimicrobials are needed to avoid these circumstances in near future. Nanomaterials offer several advantages over traditional antibiotics as effective antimicrobials, as microbes may not be able to develop resistance against these inorganic materials, therefore, it is expected that the antimicrobial activity of nanomaterials would remain same, even after multiple uses, which is one of the biggest limitations with antibiotics. Among nanomaterials, IONPs are well explored as an effective antimicrobial candidate. Studies on IONPs showed potential antimicrobial activity and literature studies revealed citrate coated Fe_3_O_4_ NPs have an inhibitory effect on *Escherichia coli, Bacillus subtilis, Candida albicans, Aspergillus niger* and *Fusarium solani* (Arakha et al. 2015; Nehra et al. 2018). Further, Patra et al. applied green synthesis for the synthesis of Fe_3_O_4_ NPs from corn plant extract and explained that NPs exerted a synergistic antibacterial and anticandidal activity (Patra et al. 2017). In another attempt, a green chemistry approach using *Couroupita guianensis* fruit extract was applied to synthesize Fe_3_O_4_ NPs and the particles exhibited potent bactericidal action on several human pathogens (Gao et al. 2017). Additionally, Ismail et al. synthesized IONPs (α-Fe_2_O_3_) and determined their antibacterial activity against Gram-positive and Gram-negative bacteria and stated that IONPs can capture *Staphylococcus aureus* through magnetic field effect (Ismail et al. 2015). Arokiyaraj et al. have developed IONPs treated with *Argemone mexicana* L. leaf extract and showed a significant colony growth inhibition against *Escherichia coli* and *Proteus mirabilis* (Arokiyaraj 2013). As discussed above, more strategies could be devised to develop synergistic IONPs platform, which can be used as a carrier system to treat microbial diseases in future.

## Summary and future perspectives

IONPs with their magnetic characteristics and contrast have already successfully applied in areas of biomedicine, including diagnostics as a probe (MRI scanning) for detection of diseases or disorders in the brain, cardiovascular, liver, blood vessels and other vital organs. Moreover, with advancements, IONPs are prepared in combinations to achieve multiple functions in a single stage like T_1_ and T_2_ MRI, and PET/CT/NIRF/MRI/PA imaging. Recently IONPs-based nanohybrids were tuned for hyperthermia and PTT, where even low concentrations are capable to enhance the heat generation at the tumor site and can be efficiently used for cellular therapies. In bio-sensing, advances in Nanohybrid IONPs synthesis paved a path to introduce enzyme mimetics, possessing peroxidase, oxidase, and catalase-like activities. In addition, the nanohybrids or conjugated NPs simplified the detection of biomolecules in a single step and laid the foundation to create novel nano-sensors and nano-devices. Further to overcome the obstacles in cancer and multi-drug resistant diseases, multifunctional IONPs were being designed for diagnosis, targeting, nanocarrier, chemo and phototherapy agents. In conclusion, more progression in the novel synthesis of nanocomposites with multifunctional modalities can find better ways to use IONPs as nano-theranostic entities in biomedicine. The future of IONPs in biomedical applications holds great promise especially in the area of disease diagnosis, early detection, cellular and deep tissue imaging, drug/gene delivery as well as multifunctional therapeutics. Although, Feraheme (US FDA approved) is being used by the consumers, more of such IONPs-based materials need to be researched and made available to the consumer market. The current emphasis of molecular medicine is to develop more novel tools, which can be used for early-stage disease diagnosis and more of point-of-care diagnostics. Integration of nanomaterials, especially IONPs, could extend the construction of the theranostic platform, which combines therapeutics with diagnostics. Such attempts would primarily make the diagnosis processes simpler, speedier and less invasive. Personalized medicine is also gaining attention and it is expected that the integration of nanotechnology could result in overarching outcomes. In coming years, multifunctional IONPs would be an attractive material for biomedical applications and may change the usual business model of pharmaceutical industries.
